# Retraction: *MicroRNA-302a* Functions as a Putative Tumor Suppressor in Colon Cancer by Targeting *Akt*

**DOI:** 10.1371/journal.pone.0139497

**Published:** 2015-09-25

**Authors:** The PLOS ONE Editors

It has been brought to the attention of the *PLOS ONE* editors that there is substantial overlap in content between this article and another article published in *PLOS ONE* by different authors: "MicroRNA-302a Suppresses Tumor Cell Proliferation by Inhibiting *AKT* in Prostate Cancer" (DOI: 10.1371/journal.pone.0124410) [2].

There is a substantial overlap in the text, and multiple figures appear similar or identical despite labels and legends indicating that the data were obtained from different cell and tissue types (prostate cancer cell lines and tissues in the above-mentioned article versus colon cancer cell lines and tissues in the retracted article).

Our follow-up in relation to how the overlap with the article by Zhang et al. (DOI: 10.1371/journal.pone.0124410) [2] arose is ongoing. Upon our follow-up with the authors of the current article by Sun et al., we have been unable to obtain the underlying data and approval documents related to the study, and as a result, the *PLOS ONE* editors have been unable to verify the integrity of the work. In light of these concerns, the *PLOS ONE* editors retract this article.
